# An Agent Framework for Land Resource Monitoring and Analysis

**DOI:** 10.3390/s26206371

**Published:** 2026-10-09

**Authors:** Xiangyu Yang, Xinxin Gao, Ying Zhang, Li Liu, Yuanfei Chang

**Affiliations:** 1 Aerospace Information Research Institute, Chinese Academy of Sciences, Beijing 100094, China; yangxiangyu23@mails.ucas.ac.cn (X.Y.);; 2 University of Chinese Academy of Sciences, Beijing 100049, China; 3 Dalian Scientific Test and Control Technology Institute, Dalian 116013, China

**Keywords:** land resource monitoring, remote sensing applications, large language models, agents

## Abstract

This paper proposes an agent framework for land resource monitoring and analysis. The framework organizes and encapsulates domain knowledge and operational workflows into standardized skill units, enabling the agent to perform task planning, execution, and reflection under skill-based constraints and thereby improving the procedural consistency of complex operational tasks. In addition, a task-semantics-based result verification module is introduced to assess the semantic consistency of intermediate results and further identify latent errors in which tools execute successfully but the resulting outputs deviate from user requirements. Experimental results show that the proposed method outperforms the ReAct, Plan-and-Execute, and Plan-and-React baselines overall, achieving a final result accuracy of 93.33% ± 1.44%. Ablation experiments demonstrate that skill-based constraints, progressive loading, and result verification all improve agent execution reliability. A dedicated evaluation of the result verification module further shows that it can effectively identify latent execution errors, achieving a Precision of 93.55%, a Recall of 90.63%, and a false-positive rate of 6.25%.

## 1. Introduction

Satellite remote sensing, with its broad spatial coverage and capability for periodic repeat observations, has become an important technical means for land resource surveys and dynamic monitoring [1]. As the demands of land resource monitoring have gradually expanded from surface change detection to comprehensive analysis and regulatory decision-making, practical workflows are no longer limited to information extraction from individual remote sensing images. Instead, they increasingly require the coordinated use of multi-source spatiotemporal data together with a series of subsequent processing procedures, such as spatial relationship analysis and statistical evaluation [2,3]. These workflows typically involve numerous steps and long processing chains, in which tool selection, parameter configuration, and execution order must be determined according to task objectives, data conditions, and operational rules. Such processes therefore rely heavily on expert experience, making it difficult for non-expert users to independently construct appropriate analytical workflows based on their task requirements.

Against this background, large language models (LLMs) and agent technologies provide a new technical pathway for bridging this expertise gap. LLMs represented by GPT [4], Qwen [5], and LLaMA [6] have been widely applied to tasks such as question answering, code generation, and text generation owing to their strong capabilities in semantic understanding and knowledge reasoning [7,8,9]. An agent is an entity capable of perceiving its environment, making autonomous decisions, and taking actions [10]. When integrated with LLMs, agents gain strong natural language processing capabilities, enabling them to interpret user instructions expressed in natural language and invoke appropriate tools to accomplish tasks, thereby demonstrating considerable potential for complex domain-specific applications [11]. In recent years, a growing number of studies have explored the application of agents in remote sensing and geographic information science. For example, Zhuang et al. developed an LLM-driven intelligent geographic information interaction system that enables natural language-based GIS tool invocation and task execution [12]. Qi et al. proposed a tool-retrieval-enhanced multi-agent framework for geographic information tasks, improving the understanding and execution of complex geospatial tasks through task parsing, workflow construction, tool retrieval, and execution modules [13]. Weiss et al. proposed MapBot, which supports operations such as data uploading, annotation, and querying through natural language dialogue and map-based interaction [14]. Jin et al. developed GeoAgent, which achieves accurate geolocation through reinforcement learning [15]. Yu et al. proposed the Plan-and-React agent framework, which combines global task planning with stepwise reactive execution to improve workflow organization and runtime error recovery in complex spatial analysis tasks [16].

Despite the demonstrated potential of these studies in task understanding, workflow planning, and tool invocation for remote sensing and geographic information applications, several limitations remain. First, land resource monitoring is highly dependent on domain-specific knowledge. Existing agents primarily rely on the general knowledge encoded in LLMs for task understanding and planning, which may lead to analytical workflows that do not conform to domain-specific operational standards [17]. Second, current research on agent reflection and self-correction mainly addresses cases with explicit error feedback, such as tool invocation failures, missing parameters, or invalid parameter formats [18], with the primary objective of ensuring successful tool execution. In contrast, insufficient attention has been paid to latent errors in which tools execute successfully but the resulting outputs deviate from the intended task semantics. For example, incorrect specification of spatial relationships such as intersection, containment, or proximity, or the direct execution of spatial operations on datasets with inconsistent coordinate reference systems, may produce formally valid outputs with incorrect operational meanings. Such problems generally do not trigger program exceptions and may therefore be mistakenly accepted by the agent as correct results, thereby compromising the reliability of subsequent analytical procedures and monitoring conclusions.

Accordingly, this study focuses on how to enable LLM-based agents to consistently generate domain-appropriate workflows, invoke tools correctly, and perform process-level quality control on intermediate results in complex land resource remote sensing monitoring tasks. The main contributions of this study are as follows: (1) domain-specific skills for land resource remote sensing monitoring are constructed by encapsulating professional domain knowledge into reusable skill units; (2) an agent framework for land resource monitoring and analysis is developed, in which domain-specific skills provide knowledge support for the coordinated processes of task planning, execution, result verification, and reflection and revision, enabling autonomous transformation of natural language requirements into professional remote sensing analysis workflows; and (3) a task-semantics-based result verification module is proposed, which extracts task intent and factual information from execution results and performs consistency diagnosis to identify latent errors where tools execute successfully but the resulting outputs deviate from user requirements.

## 2. Methodology

This study proposes an agent framework for land resource remote sensing monitoring and analysis. The framework employs a large language model as the core component for semantic understanding and decision control, integrates expert knowledge as domain-specific capability support, automatically constructs analytical workflows through skill-constrained task planning and tool invocation, and uses execution result verification together with reflection and revision for process-level quality control. The overall architecture is shown in Figure 1.

### 2.1. Construction and Progressive Loading of Domain-Specific Skills

To enhance the adaptability of large language models to domain-specific tasks, existing knowledge augmentation approaches mainly fall into two categories: model fine-tuning and retrieval-augmented generation. Model fine-tuning injects domain knowledge into model parameters through additional training. This process typically requires high-quality domain-specific training data and incurs additional training costs; moreover, model adaptation may need to be repeated when domain knowledge is updated, resulting in relatively high costs for data preparation, computation, and maintenance [19]. Retrieval-Augmented Generation (RAG) dynamically supplements model knowledge using external knowledge bases. However, conventional RAG generally returns a limited set of Top-k results according to query relevance, leaving the model to passively use only the retrieved content and making it difficult to perceive the overall structure of the knowledge base or information that has not yet been retrieved. Recent studies have further explored organizing document knowledge into hierarchical skill structures, allowing agents to navigate progressively and acquire relevant content as needed [20]. Such approaches primarily address the organization and access of knowledge content. However, land resource monitoring tasks involve explicit workflow dependencies, conditional decision logic, and operational constraints; merely retrieving relevant knowledge fragments is insufficient to fully represent the execution relationships among knowledge elements and the conditions under which they apply.

Therefore, drawing on the Agent Skills paradigm [21], this study organizes relevant domain knowledge into domain-specific skills that can directly support land resource remote sensing monitoring and analysis tasks and adopts a progressive loading mechanism that allows the agent to access information hierarchically and on demand. This design enables efficient use of the context window while preserving a global view of available knowledge, thereby providing knowledge support for task planning, execution, and reflection, as illustrated in Figure 2.

Domain-specific knowledge for land resource monitoring is typically derived from technical guidelines, policy documents, academic publications, typical cases, and other sources. Because these sources differ substantially in both representation and knowledge granularity, they cannot be directly used as task guidance for an agent. Accordingly, this study adopts a task-oriented knowledge organization strategy to transform raw domain knowledge into skills. First, skill boundaries are defined according to operational objectives, and knowledge capable of independently supporting a specific type of professional monitoring task is organized as an individual skill topic, such as cultivated land occupation, river encroachment, forest damage, or illegal construction. On this basis, operational requirements with explicit sequential relationships in technical specifications and operational workflows are transformed into standard execution procedures and inter-step dependencies within the skill. Thresholds, spatial relationships, and decision criteria defined in industry standards are converted into operational constraints applied during task execution. In addition, operational manuals, typical cases, executable scripts, exception-handling guidelines, and output templates that may need to be consulted or invoked during execution are retained as supporting resources. Through this process, fragmented domain knowledge is reorganized into professional skills that can be invoked by the agent.

To reduce the occupation of the LLM context window by large volumes of domain knowledge and minimize interference from irrelevant information during task reasoning, each skill is organized into three layers: a metadata layer, a core instruction layer, and a resource layer, which are loaded by the agent on demand according to the current task. The k-th domain-specific skill is represented as

(1) $$s_{k}=\left(m_{k},c_{k},r_{k}\right),$$

where $m_{k}$ denotes the metadata layer, $c_{k}$ denotes the core instruction layer, and $r_{k}$ denotes the resource layer.

The metadata layer stores lightweight information such as the skill identifier, skill name, and functional description. This information is loaded collectively during system initialization, enabling the agent to establish a global index of available domain capabilities and perform skill routing accordingly. Once a skill is matched to the current task, its core instruction layer is loaded, introducing the corresponding standard analytical workflow and operational constraints into the current context to guide task planning and execution. The resource layer centrally stores reference materials required for in-depth analysis and complex interpretation. Rather than being loaded in its entirety, these resources are accessed during execution when the corresponding step is reached or when a specific condition is encountered, as directed by the core instructions. This on-demand loading strategy allows domain knowledge to support the entire reasoning process while reducing context length and limiting interference from task-irrelevant information.

### 2.2. Task Planning Module

The task planning module is responsible for transforming the user’s natural language requirements into an executable structured solution. At the beginning of a task, the agent reads the metadata of all available skills and evaluates the semantic match between the user requirement and each skill metadata entry to select the skill most relevant to the current task:

(2) $$s^{*}=\underset{s_{k}\in S}{\mathrm{argmax}}Match\left(q,m_{k}\right)$$

where $S=\left\{s_{1},s_{2},\ldots ,s_{n}\right\}$ denotes the skill repository, q denotes the user requirement, $m_{k}$ denotes the metadata of the k-th skill, and Match(⋅) denotes the semantic matching process performed by the LLM between the user requirement and the skill metadata. After the target skill is identified, the agent loads its core instructions and adapts the user requirement under the constraints of the selected skill to generate a structured execution plan P, which consists of the skill identifier and a list of subtasks:

(3) $$P=\left(s_{id}^{*},\{\left(d_{i},t_{i}\right){\}}_{i=1}^{m}\right)$$

where $s_{id}^{*}$ denotes the identifier of the selected skill, $d_{i}$ denotes the description of the i-th subtask, and $t_{i}$ denotes the tool associated with that subtask. By incorporating domain-specific knowledge as constraints, the generated solution is better aligned with operational logic, thereby improving the procedural consistency and executability of long-horizon remote sensing analysis tasks.

### 2.3. Task Execution Module

The task execution module is responsible for translating the task steps generated during planning into concrete analytical operations and interacting with external data and computing environments. For each subtask $\left(d_{i},t_{i}\right)$, the agent first obtains three types of contextual information: (1) the domain-specific knowledge $r_{i}^{*}$ relevant to the current step; (2) the structured specification $f_{i}$ of the target tool $t_{i}$; and (3) the execution history summary $H_{i-1}$ of the preceding steps. The agent then generates structured tool invocation parameters based on this context:

(4) $$\theta_{i}=\mathrm{GenParam}\left(d_{i},f_{i},r_{i}^{*},H_{i-1}\right)$$

During execution, the agent sequentially invokes the corresponding analytical tools according to the execution plan and obtains the data outputs and execution status produced at each step. For the i-th task step, the execution process is expressed as

(5) $$o_{i}=\mathrm{Execute}\left(t_{i},\theta_{i}\right)$$

where $t_{i}$ denotes the analytical tool invoked at the current step, $\theta_{i}$ denotes its input parameters, and $o_{i}$ denotes the resulting output. In this manner, the LLM is responsible for parameter generation and tool invocation, whereas the actual spatial computations are performed by specialized analytical tools, thereby reducing the uncertainty associated with direct computation by the LLM.

### 2.4. Task-Semantics-Based Result Verification Module

Existing agents primarily focus on whether tool invocation is successful. However, successful tool execution does not necessarily indicate that the analytical results satisfy user requirements. Even when input parameters are valid and no program errors occur, a tool may produce results whose geometry type, spatial relationship, or statistical meaning is inconsistent with the task objective. To identify such errors that are difficult to capture through program exceptions, this study proposes a task-semantics-based result verification module.

Based on the original user requirement and the description of the current subtask, the system uses the LLM to infer the task semantics of the current step. The fields used for task semantic extraction are shown in Table 1. Through this structured representation, spatial semantic requirements originally implicit in natural language are transformed into explicit and verifiable constraints.

After obtaining the task semantics, the agent further extracts factual information from the tool execution results. Unlike semantic extraction, execution result inspection does not rely on subjective judgments by the LLM. Instead, scripts running in the QGIS environment extract the relevant information, write the inspection results to a JSON file, and return them to the agent. Depending on the type of tool invoked, the system extracts task-relevant information such as geometry type, area, feature count, and coordinate reference system.

Once the task semantics $I_{i}$ and execution result facts $F_{i}$ are obtained, the system treats the task semantics as the constraints that the current step is expected to satisfy and the execution result facts as the objective state after tool execution. It then determines whether the result satisfies the spatial semantic requirements of the current subtask. The diagnostic function is expressed as

(6) $$\Delta_{i}=Diagnose\left(I_{i},F_{i}\right)$$

where $\Delta_{i}$ denotes the verification result, consisting of error warnings and revision suggestions. This mechanism enables the effective detection of errors such as inappropriate coordinate reference systems, misassignment of layer roles, and misuse of spatial relationships, thereby providing more reliable process-level quality control for remote sensing agents.

### 2.5. Reflection and Revision Module

Although domain-specific knowledge is incorporated during task planning to generate an appropriate workflow, deviations from task objectives may still occur during execution because of complex task environments, changing data conditions, and the inherent uncertainty of LLM reasoning.

Therefore, this study introduces a reflection and revision module that enables the agent to retrieve relevant knowledge and adapt its subsequent actions according to the current task plan, tool execution status, and verification results. Four types of actions are supported: continuing execution, adjusting tool parameters, selecting an alternative tool, and inserting necessary steps. To prevent ineffective iterative loops, a maximum number of retries is specified for each subtask. If a correct result cannot be obtained after multiple revision attempts, the agent terminates the current execution chain and returns the available results.

## 3. Experiments

### 3.1. Experimental Setup

The experiments were designed around the proposed agent framework and its key mechanisms and were conducted at three levels: overall execution performance, contributions of key mechanisms, and result verification capability. The overall execution performance experiment evaluates the overall capability of the proposed method to execute complex land resource monitoring tasks and compares it with ReAct, Plan-and-Execute, and Plan-and-React. The key-mechanism experiments assess the effects of progressive loading, domain-specific skills, and the result verification module on agent performance. Finally, a dedicated evaluation of the result verification module was conducted by constructing error cases involving coordinate reference system errors, layer-role swaps, and misuse of spatial predicates to assess its error detection capability.

#### 3.1.1. Experimental Environment and Tool Configuration

The experiments were conducted on Windows 11. The agent framework was implemented using LangChain 0.3, and DeepSeek-V4-Pro-0813 was used as the base reasoning model via DeepSeek’s official API. The model’s thinking mode was enabled, with the reasoning effort set to high. The top_p parameter was set to 1, and max_tokens was set to 64K. To minimize the effects of differences in the base model, tool configuration, and runtime environment on the experimental results, all experimental groups used identical model inference settings, task instructions, experimental data, tool sets, and runtime environments. For methods that support reflection or replanning, the maximum number of retries for each subtask was uniformly set to three. Considering the stochasticity of LLM inference, each experimental configuration was independently repeated three times, with the full set of test tasks executed in each run. To ensure experimental traceability, the system automatically recorded the execution process of each run, including the selected skill, structured execution plan, tool calls and generated parameters, result verification reports, reflection and revision decisions, and final responses. All evaluation metrics are reported as mean ± SD over the three runs.

To support the complete execution of land resource remote sensing monitoring tasks, the experimental environment was equipped with 28 callable tools, all uniformly encapsulated using the Model Context Protocol (MCP). The agent obtains tool functionality and parameter specifications from standardized tool declarations. By function, the tools are divided into four categories: data management, spatial analysis, remote sensing processing, and result statistics and reporting. The nine data management tools are mainly used to retrieve layer information, select remote sensing imagery, load and remove data, and create output directories. The 11 spatial analysis tools provide general GIS capabilities, including buffer analysis, overlay analysis, spatial joins, and coordinate reference system transformation. The five remote sensing processing tools are used for target extraction, change detection, and vegetation index calculation. The remaining three result statistics and reporting tools are used for statistical analysis and report generation.

#### 3.1.2. Study Areas and Experimental Data

To cover different geographic environments and land resource monitoring scenarios, Jiujiang in Jiangxi, Mentougou in Beijing, Zhoukou in Henan, and Jinan in Shandong were selected as the test areas. The experimental data consist of remote sensing imagery, vector constraint data, and auxiliary operational data. The remote sensing imagery primarily includes medium- to high-spatial-resolution satellite images from Jilin-1, Gaofen-2, and Sentinel-2, acquired in October 2018, November 2019, and November 2023. The agent selects the appropriate data according to the requirements of each task. In addition to remote sensing imagery, the experiments use spatial constraint and base geographic data, including river management boundaries, cultivated land protection redlines, and administrative boundaries, as well as auxiliary operational data such as points of interest (POIs), public reports, and administrative penalty records. These data are used for further information association and integrated analysis of the monitoring polygons.

#### 3.1.3. Task Benchmark Construction

To systematically evaluate the agent’s capability in long-horizon land resource remote sensing monitoring and analysis tasks, we constructed a task benchmark consisting of natural language task instructions and corresponding reference execution trajectories. The benchmark covers four typical land resource monitoring scenarios: cultivated land occupation, forest damage, river encroachment, and illegal construction. Each scenario contains 10 task instructions. The tasks differ in user requirements, natural language formulations, monitoring areas, and data combinations, thereby reducing the influence of fixed linguistic templates and a single processing workflow on the experimental results. Each test task is executed as an independent instance, with no historical dialogue or other information shared across tasks. A single task typically requires more than 10 consecutive tool invocations, allowing the planning and execution capabilities of the agent in long-horizon tasks to be evaluated.

To ensure consistent evaluation criteria, multiple experts with experience in remote sensing and geographic information analysis developed a reference execution trajectory for each task according to its requirements. Each reference trajectory specifies the necessary analytical steps, applicable tools, key parameter constraints, and final outputs required to complete the task. The reference trajectories are used to determine whether the agent omits essential steps and whether its tool invocations satisfy task requirements; the agent is not required to reproduce the reference trajectory exactly on a step-by-step basis. An execution is also considered valid when the agent completes all necessary analytical procedures using an equivalent combination of tools.

In addition to the reference execution trajectories, a final operational reference result was constructed for each test task to evaluate the agent’s final output. Using monitoring polygons as the basic units, the reference result records the clue association level for each polygon and the identifiers of associated POIs, public reports, and other operational clue objects.

#### 3.1.4. Evaluation Metrics

To objectively evaluate agent performance, the execution trajectories constructed in Section 3.1.3 are used as the reference, and performance is assessed from four perspectives: tool invocation, execution process, final results, and model overhead.

Tool Call Accuracy (TCA): This metric measures the correctness of tool selection and parameter generation during task execution. A tool call is considered correct when the selected tool can correctly accomplish the current subtask and its key parameters satisfy the task requirements.

(7) $$TCA=\frac{N_{\mathrm{correct}\_\mathrm{call}}}{N_{\mathrm{call}}}\times 100\%$$

where *N_correct_call_* denotes the number of correct tool calls, and *N_call_* denotes the total number of tool calls generated by the agent across all test tasks.

Trajectory Completeness Rate (TCR): This metric measures whether the agent executes all necessary steps required to complete a task. A trajectory is considered complete if all key steps required by the task are executed and the necessary inter-step dependencies are satisfied; the actual execution path is not required to exactly match the reference trajectory.

(8) $$TCR=\frac{N_{\mathrm{complete}\_\mathrm{task}}}{N_{\mathrm{task}}}\times 100\%$$

where *N_complete_task_* denotes the number of tasks with complete execution trajectories, and *N_task_* denotes the total number of test tasks.

Final Result Accuracy (FRA): This metric evaluates the correctness of the agent’s final operational results. It compares only the final output with the reference operational result, irrespective of the specific execution path taken by the agent. A task is considered correct only when the clue association levels and associated objects for all monitoring polygons exactly match the corresponding reference result.

(9) $$FRA=\frac{N_{\mathrm{result}\_\mathrm{correct}}}{N_{\mathrm{task}}}\times 100\%$$

where *N_result_correct_* denotes the number of tasks with correct final results.

Average Token Consumption: For each task, the total number of input and output Tokens consumed by the agent during execution is calculated, and the average across all tasks is used to measure the computational overhead of the method.

(10) $$AvgToken=\frac{1}{N_{\mathrm{task}}}\sum_{i=1}^{N_{\mathrm{task}}}\left(T_{i}^{\mathrm{in}}+T_{i}^{\mathrm{out}}\right)$$

where *T_i_^in^* and *T_i_^out^* denote the numbers of input and output Tokens, respectively, generated by all LLM calls during execution of the *i*-th task.

### 3.2. Overall Performance Comparison

To evaluate the effectiveness of the proposed method, the classical ReAct and Plan-and-Execute agent paradigms, together with the domain-specific Plan-and-React agent [17], were selected as baseline methods. ReAct follows an interleaved Reasoning–Acting paradigm, reasoning over the current state and incrementally selecting the next action. Plan-and-Execute first decomposes a task into an ordered sequence of subtasks, executes only the current step of the remaining plan at each iteration, and then replans the subsequent steps based on completed results until a final response is produced. Plan-and-React determines the overall execution workflow through global task planning and employs a reactive executor during step execution to dynamically adjust the current action according to tool execution results and environmental feedback. The experimental results are shown in Table 2.

The experimental results show that the proposed method achieves the best performance on all three metrics, TCA, TCR, and FRA, exceeding the second-best method by 6.04, 14.16, and 22.50 percentage points, respectively. These results demonstrate clear advantages in tool invocation, completeness of long-horizon execution, and the quality of final task outcomes. Across the three independent runs, the proposed method also exhibits relatively small standard deviations across all metrics. In particular, the variation in FRA is lower than that in all three baseline methods, indicating that the proposed method achieves high average performance while maintaining good execution stability. Because Plan-and-Execute attains the highest FRA among the three baselines, it was further used as the primary comparator for a statistical significance test on FRA. A two-sided Welch *t*-test shows that the difference in FRA between the proposed method and Plan-and-Execute is statistically significant (t = 7.22, *p* = 0.012 < 0.05).

Further analysis of the different methods shows that ReAct incrementally reasons over the current execution state and selects the next action, providing considerable runtime flexibility. However, it lacks global constraints over the complete task workflow and is therefore prone to omitted steps, repeated tool calls, and local decision errors in long-horizon spatial analysis tasks, resulting in relatively low TCR and FRA. Plan-and-Execute strengthens the organization of the overall task workflow by generating a complete plan in advance, thereby improving both TCR and FRA over ReAct. Plan-and-React introduces runtime reactive adjustment on top of global planning, improving adaptability to changes in the execution state. Nevertheless, frequent local adjustments may gradually cause actual execution to deviate from the original plan, leading to inconsistencies between the plan and the execution trajectory or to the accumulation of local corrections in long-horizon tasks; consequently, its overall performance remains constrained.

In terms of computational overhead, the proposed method has substantially lower average Token consumption than the three baseline agents. This is mainly because the three baseline agents need to read the complete tool documentation of all available tools at each tool execution step to support tool selection and subsequent adjustment. In contrast, the proposed method reads tool information only during the planning stage, where domain-specific skills guide task decomposition and tool selection; during subsequent execution, each step loads only the declaration of the single tool corresponding to the current subtask. This design effectively reduces the context overhead caused by repeatedly loading tool information.

### 3.3. Ablation Experiments on Key Mechanisms

To quantitatively analyze the independent contributions of the key mechanisms in the proposed agent framework, three ablation settings were constructed: w/o Skill, w/o Progressive Loading, and w/o Verification. Under w/o Skill, domain-specific skills are completely removed, and the agent cannot access any preconstructed skill metadata, core instructions, or resource content during task planning and execution; tasks are completed solely on the basis of the current task instruction and the available tools. Under w/o Progressive Loading, the same skill knowledge as in the full method is retained, but all skill knowledge is loaded at system initialization rather than being progressively loaded on demand. Under w/o Verification, the task-semantics-based result verification module is removed, and consistency between tool execution results and the semantics of the current task is no longer diagnosed, while all other planning and execution mechanisms remain unchanged. The experimental results are shown in Table 3.

The ablation results show that removing domain-specific skills decreases TCA, TCR, and FRA by 6.12, 8.33, and 4.16 percentage points, respectively. The larger reductions in TCA and TCR indicate that the standard analytical procedures, operational constraints, and operational knowledge provided by domain-specific skills effectively constrain the agent’s planning and execution processes, reducing tool misselection, omission of critical steps, and deviations from the intended execution path. The smaller decrease in FRA can be attributed to the result verification module, which can still detect and correct some errors and thereby partially compensate for the absence of skill-based constraints. Notably, even after all preconstructed skills are removed, the agent is still able to complete most test tasks. This finding suggests, on the one hand, that the result verification and reflection-and-revision mechanisms can compensate for some execution deviations and, on the other hand, that the proposed framework does not rely exclusively on predefined skills to accomplish tasks and retains strong task execution capability when no domain-specific skills are available. Removing domain-specific skills increases the average Token consumption by 2734.08 Tokens, a relatively small change. This indicates that removing skills does not reduce the overall reasoning overhead; rather, the absence of professional knowledge constraints may increase ineffective tool calls and subsequent corrections. Across the three independent runs, the standard deviations of all metrics are higher after domain-specific skills are removed than under the full method, indicating that the absence of domain-specific skills not only reduces average execution performance but may also make execution outcomes less stable across runs.

After removing the progressive loading mechanism, all task-performance metrics decrease slightly, with TCA, TCR, and FRA declining by 1.12, 1.66, and 0.83 percentage points, respectively. This is because loading the core skill instructions together with all associated resources at once introduces a large amount of information that is not directly relevant to the current task stage into the model context, increasing the difficulty for the agent to filter and assess task-relevant information and thereby introducing additional interference into the reasoning process. Considering that resource consumption alone may be affected by differences in task completion quality, this study further draws on the approach of Wang and Matsuhashi [22], which evaluates resource consumption under comparable service effectiveness. After progressive loading is removed, FRA decreases only slightly from 93.33% to 92.50%, a reduction of 0.83 percentage points, indicating that the two settings achieve comparable task completion performance. Meanwhile, the average Token consumption increases from 55,021.55 to 135,430.47, approximately 2.46 times that of the full method. These results indicate that progressive loading can substantially reduce context length and model inference overhead while largely preserving task performance, thereby improving the efficiency of skill knowledge utilization. Compared with the full method, removing progressive loading produces no substantial change in the variability in the performance metrics, which remains relatively low.

After removing the result verification module, TCA and TCR change only slightly, whereas FRA decreases from 93.33% to 88.33%, a reduction of 5.00 percentage points. This indicates that the result verification module has a relatively limited direct effect on early-stage task planning and tool selection. Its main role is manifested after tool execution, where it identifies latent errors arising from inconsistencies between intermediate results and task semantics and triggers subsequent correction, thereby reducing the probability that erroneous results propagate along the execution chain. Removing this module decreases the average Token consumption by 8384.35 Tokens, indicating that result verification introduces additional reasoning overhead but can substantially improve FRA. In terms of performance variability, the metrics remain relatively stable after the result verification module is removed.

Overall, the ablation experiments show that domain-specific skills primarily provide professional knowledge constraints for task planning and tool invocation, progressive loading mainly controls the context overhead introduced by skill knowledge, and result verification focuses on post-execution semantic consistency assessment and error correction. The three mechanisms operate at the levels of knowledge support, context management, and process-level quality control, respectively, and jointly improve the execution performance and efficiency of complex remote sensing monitoring tasks.

### 3.4. Evaluation of the Result Verification Module

The ablation experiment in the preceding section reflects the effect of the result verification module on complete task execution outcomes, but it does not directly quantify its accuracy in detecting latent errors themselves. Therefore, this section conducts a dedicated evaluation of the result verification module by constructing both correct execution results and latent-error results and measuring Precision, Recall, and the false-positive rate (FPR). The simulation experiment includes 160 latent-error samples and 160 correct results consistent with the task semantics as negative samples, yielding 320 test instances in total. The performance test results are shown in Table 4.

The results show that the result verification module can effectively identify latent execution errors. Overall Precision reaches 93.55%, Recall 90.63%, and FPR 6.25%, indicating that the module can correctly identify most latent errors during execution while maintaining a low false-positive probability for correct execution results.

Among the different error types, CRS errors achieve the best detection performance, with a Precision of 100.00%, a Recall of 97.50%, and no false positives. This is mainly because the coordinate reference system and its suitability can be determined from objective execution result information such as CRS metadata. Join-method errors achieve a Precision of 94.87% and a Recall of 92.50%, likewise demonstrating high detectability. By contrast, layer-role swaps and spatial-relation errors show relatively lower Precision and Recall, and both have an FPR of 10.00%, indicating greater detection difficulty. Layer-role errors are primarily associated with task-intent parsing: the LLM may incorrectly assign the roles of the input and join layers during the association process, leading to false negatives or false positives. Spatial-relation errors mainly arise from insufficient distinction among the semantic boundaries of different spatial predicates, which can cause errors when specific spatial constraints are extracted from natural language task descriptions.

### 3.5. Case Study

To further demonstrate the practical applicability of the proposed method in land resource monitoring tasks, a forest damage monitoring task was selected as a case study. The task involves multi-source data, including remote sensing imagery, POIs, and public reports, and requires the agent to perform a sequence of operations, including image selection, data loading, object extraction, spatial analysis, statistical analysis, and report generation. The final results and the agent execution process are presented to users through visualization, as shown in Figure 3.

## 4. Conclusions

This study proposes an agent framework for land resource remote sensing monitoring and analysis. The framework consists of three coordinated components: domain-specific skills for land resource monitoring and analysis, skill-constrained task planning and execution, and execution result verification with reflection and revision. Together, these components enable the automatic construction and execution of remote sensing and geospatial analysis workflows from natural language task requirements. The overall performance comparison shows that, under a unified base-model setting, the proposed method outperforms ReAct, Plan-and-Execute, and Plan-and-React in Tool Call Accuracy (TCA), Trajectory Completeness Rate (TCR), and Final Result Accuracy (FRA). Moreover, the results of three independent experimental runs exhibit only small variations, indicating that the framework achieves both strong execution performance and stable behavior. The ablation experiments further show that domain-specific skills mainly improve the procedural consistency of task planning and execution by providing standard analytical workflows, operational constraints, and domain-specific operational knowledge. Progressive loading substantially reduces context length and Token consumption while largely preserving task performance. The result verification module primarily contributes to process-level quality control after tool execution by identifying latent errors in intermediate results and thereby improving FRA. A dedicated evaluation of the result verification module further demonstrates an overall detection Precision of 93.55% for latent execution errors, confirming its effectiveness in identifying errors related to coordinate reference systems, swapped layer roles, incorrect join methods, and spatial relationships. This study is not primarily intended to automate generic GIS workflows. Rather, it addresses the need for coordinated execution across multiple stages of land resource remote sensing monitoring, including remote sensing data processing, extraction of surface objects and change information, multi-source spatial information association and analysis, and monitoring-result generation. To this end, remote sensing processing capabilities, geospatial analysis capabilities, and domain-specific knowledge are integrated within a unified agent framework. The proposed framework can autonomously organize remote sensing processing and subsequent spatial analysis according to task requirements, thereby improving the automation and reliability of transforming remote sensing observations into operational analysis products and providing a new approach to the intelligent execution of complex remote sensing monitoring tasks.

## Figures and Tables

**Figure 1:**
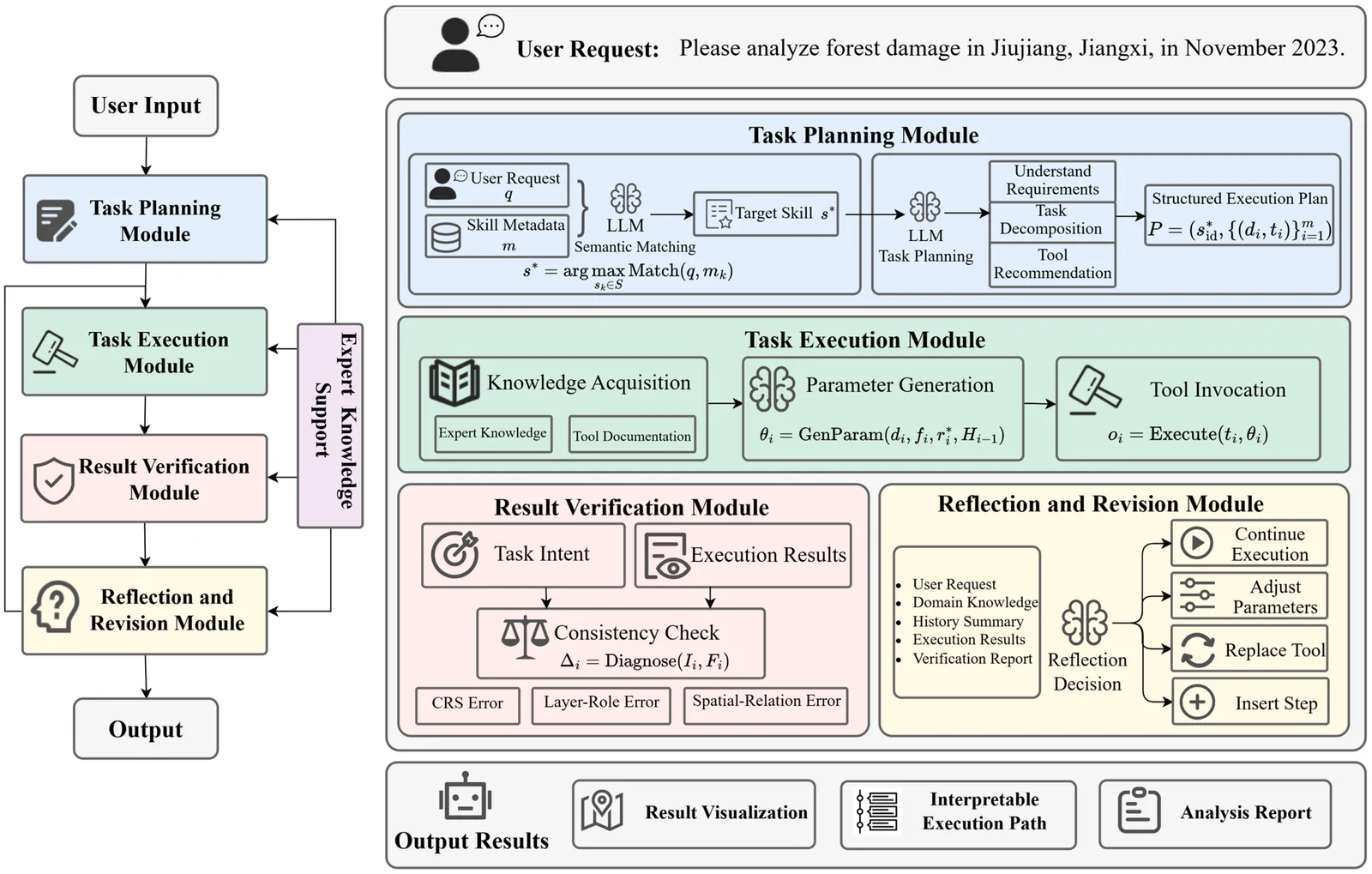
Agent framework for land resource monitoring and analysis.

**Figure 2:**
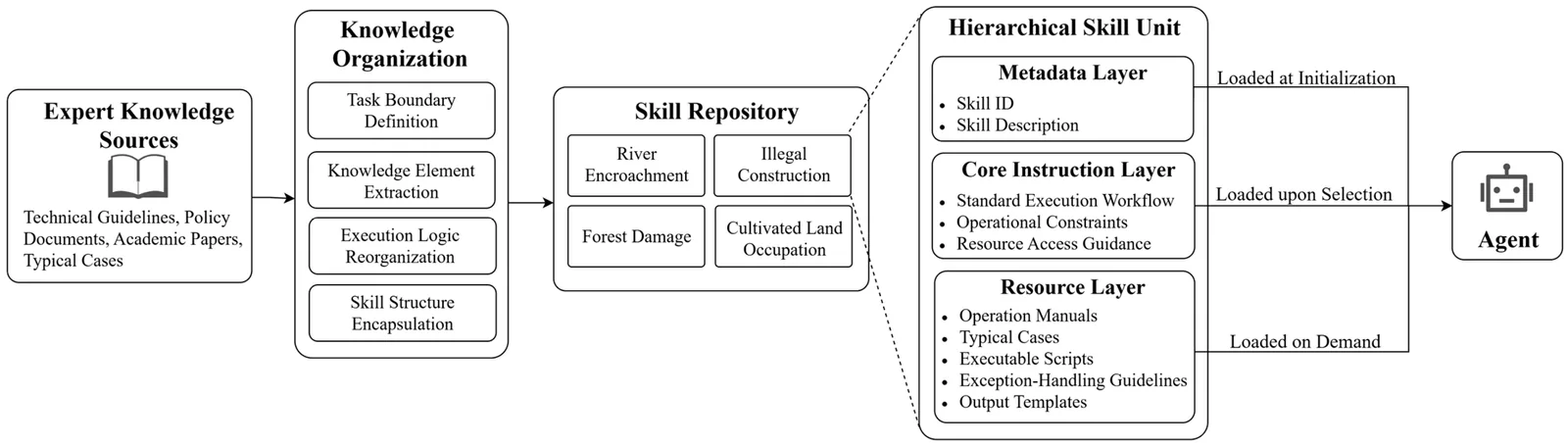
Domain-specific professional skill construction method.

**Figure 3:**
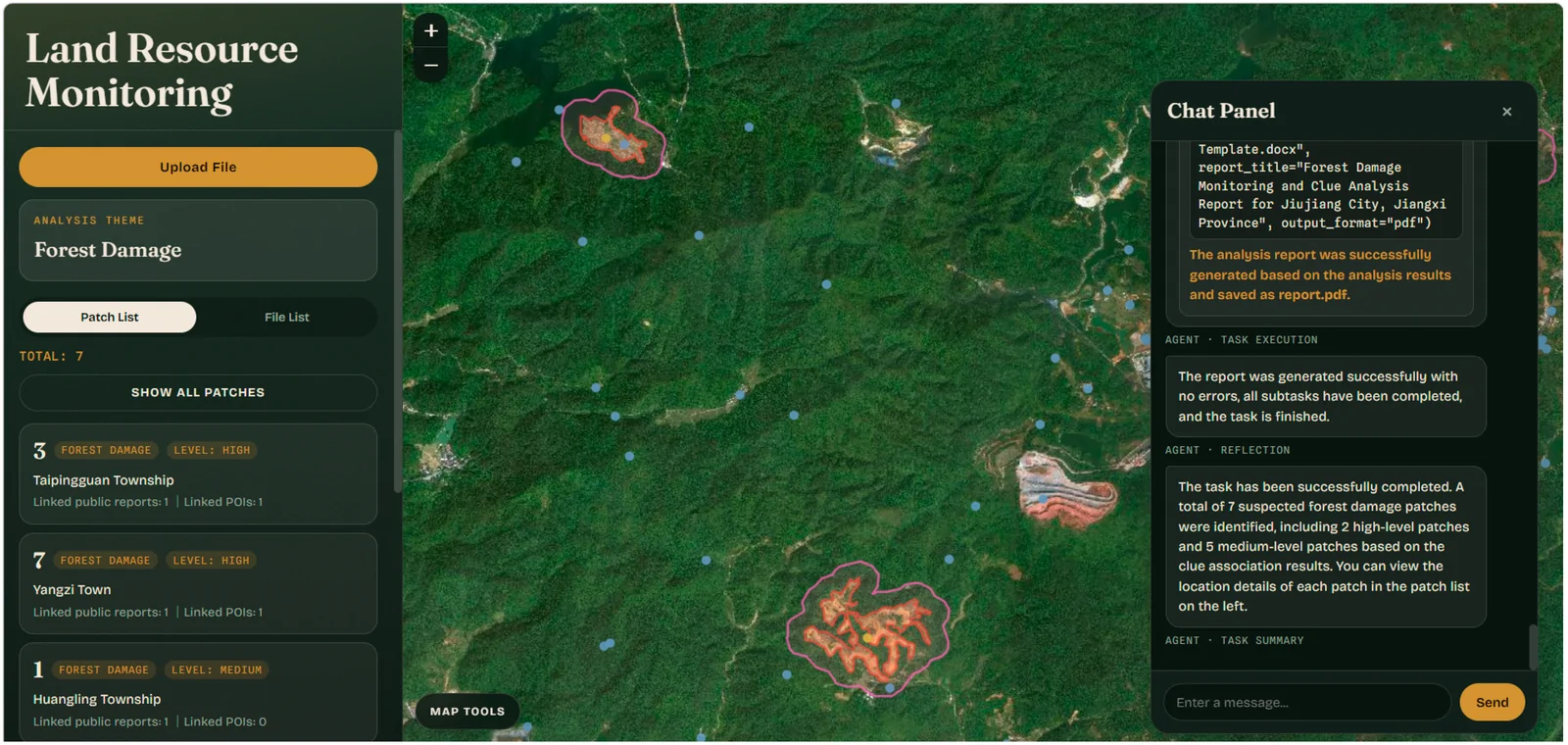
Visualization of forest damage monitoring results.

**Table 1:** Example fields for task semantic extraction.

Field	Meaning	Example Values
output_geometry	Expected output geometry	point/line/polygon/null
primary_layer_role	Role of the primary analysis layer	remote_sensing_patches/POI/null
primary_geometry	Geometry of the primary analysis layer	point/line/polygon/null
secondary_layer_role	Role of the associated or constraint layer	POI/protection redline/administrative boundary/null
secondary_geometry	Geometry of the associated or constraint layer	point/line/polygon/null
distance_m	Distance constraint (m)	50/100/200/null
spatial_relation	Spatial relationship	intersects/within/contains/null
join_mode	Spatial join mode	one_to_one_first/one_to_one_max/one_to_many/null
discard_unmatched	Whether unmatched features are discarded	true/false/null

**Table 2:** Results of baseline comparison experiments.

Method	TCA/%	TCR/%	FRA/%	Avg. Token
ReAct	88.65 ± 1.17%	75.00 ± 6.61%	65.83 ± 6.29%	147,718.67 ± 7565.35
Plan-and-Execute	90.24 ± 0.71%	81.67 ± 3.82%	70.83 ± 5.20%	129,983.63 ± 5334.47
Plan-and-React	90.79 ± 1.07%	80.83 ± 2.89%	70.00 ± 4.33%	124,934.35 ± 6049.32
Ours	96.83 ± 0.96%	95.83 ± 2.89%	93.33 ± 1.44%	55,021.55 ± 2292.79

**Table 3:** Results of ablation experiments.

Method	TCA/%	TCR/%	FRA/%	Avg. Token
Ours	96.83 ± 0.96%	95.83 ± 2.89%	93.33 ± 1.44%	55,021.55 ± 2292.79
w/o Skill	90.71 ± 1.19%	87.50 ± 4.33%	89.17 ± 3.82%	57,755.63 ± 3064.26
w/o Progressive Loading	95.71 ± 0.95%	94.17 ± 2.89%	92.50 ± 2.50%	135,430.47 ± 2980.02
w/o Verification	96.43 ± 0.71%	94.17 ± 1.44%	88.33 ± 2.89%	46,637.20 ± 2372.24

**Table 4:** Performance Test Results of the Result Verification Module.

Error Type	Precision/%	Recall/%	FPR/%
CRS errors	100.00	97.50	0.00
Layer-role swaps	89.74	87.50	10.00
Join-method errors	94.87	92.50	5.00
Spatial-relation errors	89.47	85.00	10.00
Overall	93.55	90.63	6.25

## Data Availability

The raw data supporting the conclusions of this article will be made available by the authors on request.
